# Effects of Ion Beam Etching on the Nanoscale Damage Precursor Evolution of Fused Silica

**DOI:** 10.3390/ma13061294

**Published:** 2020-03-13

**Authors:** Yaoyu Zhong, Yifan Dai, Feng Shi, Ci Song, Ye Tian, Zhifan Lin, Wanli Zhang, Yongxiang Shen

**Affiliations:** Laboratory of Science and Technology on Integrated Logistics Support, College of Intelligence Science and Technology, National University of Defense Technology, 109 Deya Road, Changsha 410073, Hunan, China; dyf@nudt.edu.cn (Y.D.); sf.wind@yahoo.com (F.S.); sunicris@163.com (C.S.); tianyecomeon@sina.cn (Y.T.); std_lzf@163.com (Z.L.); zhangwanli17@nudt.edu.cn (W.Z.); 15580949450@163.com (Y.S.)

**Keywords:** fused silica, ion beam etching, nanoscale defect, laser damage resistance, surface quality

## Abstract

Nanoscale laser damage precursors generated from fabrication have emerged as a new bottleneck that limits the laser damage resistance improvement of fused silica optics. In this paper, ion beam etching (IBE) technology is performed to investigate the evolutions of some nanoscale damage precursors (such as contamination and chemical structural defects) in different ion beam etched depths. Surface material structure analyses and laser damage resistance measurements are conducted. The results reveal that IBE has an evident cleaning effect on surfaces. Impurity contamination beneath the polishing redeposition layer can be mitigated through IBE. Chemical structural defects can be significantly reduced, and surface densification is weakened after IBE without damaging the precision of the fused silica surface. The photothermal absorption on the fused silica surface can be decreased by 41.2%, and the laser-induced damage threshold can be raised by 15.2% after IBE at 250 nm. This work serves as an important reference for characterizing nanoscale damage precursors and using IBE technology to increase the laser damage resistance of fused silica optics.

## 1. Introduction

Optical materials, such as fused silica, are widely applied in high-power inertial confinement fusion laser systems. Many studies have been conducted on the optical defects generated from the fabrication of fused silica because of their importance. These optical defects in fused silica will evolve into laser damage precursors and seriously lead to laser damage because of the illumination of sufficient fluence ultraviolet lasers [1,2,3,4]. Numerous studies have revealed that contamination (metal impurities and redeposited compounds) and fracture defect (brittle scratches) are the main optical defects in the polishing and mitigation of fused silica optics. An increasing number of researchers have realized that microscale and nanoscale optical defects are the important barriers in improving laser-induced damage threshold (LIDT) and could lead to laser damage [5,6]. Therefore, the optical defects in the fabrication of fused silica optics for high-power laser systems should be suppressed and mitigated. To date, hydrofluoric (HF) acid chemical etching is widely used to mitigate optical defects. Subsurface fracture defects can be corroded and metal contamination becomes soluble in HF acid, which are significant in raising the LIDT of fused silica [7]. Lawrence Livermore National Laboratory utilizes “advanced mitigation process” in the postprocessing of high fluence laser optics at the National Ignition Facility (NIF) [8]. However, a serious risk is found where the roughness of fused silica surface will be destroyed in deep HF acid chemical etching. The surface can be contaminated with microscale and nanoscale residual precipitations [5,9,10].

The inadequacies of the current HF acid chemical etching have been recognized, thereby requiring the development of new approaches. Elastic magnetorheological finishing (MRF) could lead to fused silica postprocessing prior to HF acid chemical etching. MRF can remove fracture defects and obtain a nonfracture subsurface during operation [11,12]. The fused silica surface will be heavily contaminated with iron caused by the carbonyl iron powder in the MRF fluid. Then, shallow HF acid chemical etching is essential to remove the contaminants from the fused silica [13]. Some researchers have developed a practical technology called reactive ion etching (RIE) by combining dynamic HF chemical etching to compensate for the shortage of HF acid chemical etching for fused silica. The LIDT of fused silica surface immensely improves through the treatment of 1 μm RIE and 3 μm HF etching [14,15]. The removal amount is immensely reduced, and high surface precision is ensured when improving laser damage resistance using the two optimized technologies. Although these combined techniques can effectively reduce the etching depth, they cannot overcome the limitations of HF acid chemical etching.

Noncontact ion beam etching (IBE) can provide a nanometer/sub-nanometer precision fabrication technology for fused silica because of the ion sputtering effect. It plays an important role in determining the ultraprecision of lithography and large telescope optics [16,17]. However, the research on the low-damage fabrication of fused silica optics using IBE is still in the initial stage. Compared with the above combined techniques, IBE does not introduce iron contamination or residual precipitations into the fused silica surface. A peer-reviewed research has shown that IBE can remove polishing residual contamination from the surface of optics to improve LIDT while maintaining surface precision to obtain a supersmooth surface [18,19,20]. As a potential nanometer precision postprocessing technology, IBE can be utilized to mitigate nanoscale damage precursors in the present study [18]. However, many important aspects of IBE for mitigating the nanoscale damage precursors of fused silica remain controversial. Nanoscale damage precursors mainly include impurity contamination and structural defects hidden or beneath the polished redeposition layer. The evolution of impurity contamination and structural defects during IBE is crucial. The details on the nanoscale damage precursor evolutions remain inadequate. Thus, many characterization results of the ion-etched surface should be presented, and considerable efforts should be exerted to achieve accurate analysis through mutual corroboration in the experiments of ion beam-etched surfaces. Understanding the mitigation mechanisms of IBE is valuable in improving the laser damage resistance.

This paper aims to determine the characteristics and evolution of nanoscale damage precursors, such as the contamination and chemical structural defects, during IBE. Section 2 introduces the sample preparation and experimental design. Section 3 presents the measurement results and analysis. Section 4 discusses the influences of measurement results on the laser damage resistance of fused silica optics. Section 5 provides the conclusions.

## 2. Sample Preparation and Experimental Design

Six commercial fused silica samples are marked as #0, #1, #2, #3, #4, and #5. The sizes of the prepared samples are all 50 mm × 50 mm × 10 mm. Samples are prepared by LANGUANG Optical Technology CO., LTD (Dongguan, China). They are treated through conventional polishing, which effectively avoids the densification effect that occurs in the material removal process and simultaneously removes the subsurface damage.

The surfaces of samples #0, #1, #2, #3, #4, and #5 are etched at 0, 50, 100, 150, 200, and 250 nm depths using ion beam, respectively. IBE experiments are performed using a self-developed IBE machine. The process parameters are fixed at a beam energy of *E*_ion_ = 900 eV and beam current of *J*_ion_ = 6 mA, and the bombardment of Ar^+^ ions is at normal incidence. The removal rate of fused silica is approximately 11.7×10^−3^ mm^3^/min. IBE does not produce the polishing redeposition layer, and the characteristics of nanoscale damage precursors and surface morphology at different ion beam etched depths can be directly measured.

Time-of-flight secondary ion mass spectrometry (TOF-SIMS, ULVAC-PHI, Kanagawa, Japan) is conducted on samples #0 and #5 to characterize the change in impurity contamination distribution on the IBE surface. A fluorescence spectrometer (JY TAU-3, JY, Paris, France) and a confocal Raman spectrometer (Bruker Senterra, Bruker, Billerica, USA) are applied to provide the spectral information of measured zones on the six surfaces for characterizing the surface structural defects. Cross-sectional high-resolution transmission electron microscopy (HRTEM, FEI Talos F200X, FEI, Hillsborough, USA) experiments are conducted to observe the subsurface nanoscale damage evolution on samples #0 and #5 before and after IBE. A photothermal absorption detection system (PTS-2000-RT-C, ZC Optoelectronic Technology, Hefei, China) and LIDT measurement are both used on six samples to evaluate the laser damage resistance of different IBE surfaces.

## 3. Results

### 3.1. Evaluation of Impurity Contamination Concentration

Impurity contamination in the polishing redeposition layer is deeply influenced by polishing conditions, such as abrasive powder. The impurity elements in the polishing redeposition layer on a traditional polished surface mainly contain Ce, Fe, and Al [21]. TOF-SIMS is conducted on the original sample and the IBE 250 nm sample to characterize the impurity contamination distribution change on the IBE surface. TOF-SIMS is a relative measurement, and no absolute calibration for element content is available. The analysis areas in the center of two samples are randomly selected in the test. The analysis conditions used in TOF-SIMS are described as follows: a gallium source is used as the analysis ion and the initial ion sources, the ion beam energy is 25 keV, the analysis area is 500 μm × 500 μm, and the acceleration voltage is 5 kV.

**Figure 1:**
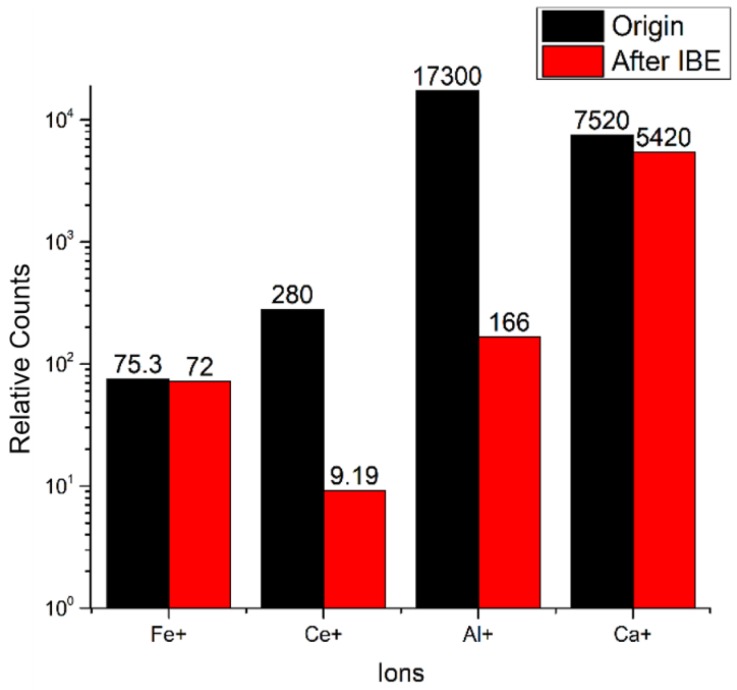
The relative concentration of metal impurities detected by time-of-flight secondary ion mass spectrometry (TOF-SIMS).

**Figure 2:**
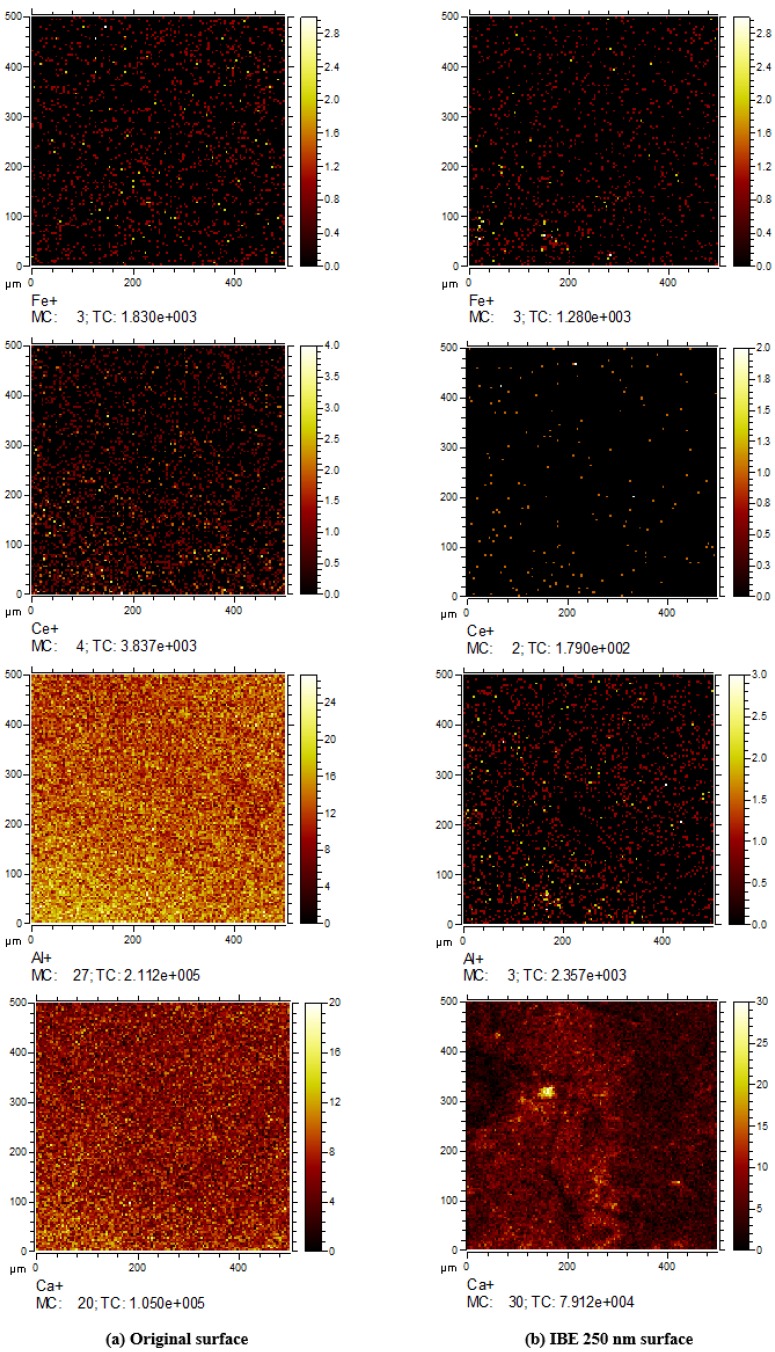
The concentration distribution of metal impurities detected by TOF-SIMS. (**a**) Original surface: the surface is enriched in Fe, Ce, Al and Ca element; (**b**) Ion beam etching (IBE) 250 nm surface: The concentration distributions of Ce, Al, and Ca become sparse. However, the concentration distribution of Fe changes little, and some bright spots are enriched in the Ca element.

Figure 1 shows the relative concentration of surface metal impurity detected by TOF-SIMS during IBE. Figure 2 characterizes the concentration distribution of surface metal impurity on samples #0 and #5 before and after IBE through TOF-SIMS. As shown in Figure 1, each ion intensity is normalized to total ion intensity, and the value is ×10^6^. On the traditional polished surface (sample #0), all the distributions of Fe, Ce, Al, and Ca, which are detected to probably cause strong absorption in the UV, are extremely uniform. For IBE 250 nm (sample #5), the surface metal impurity concentration decreases. The concentration of Ce is immensely reduced to at least 96.7% of the original. The concentration of Al decreases by approximately 99.0% of the original. The Ca element is enriched in the testing area, although its concentration slightly decreases by approximately 27.9%. The concentration distributions of Ce, Al, and Ca become sparse. However, there are still some bright spots in the distribution picture of the Ca element in Figure 2. It indicates that the bright spots on the surface are enriched in Ca element. The distribution of the Ca element may be deeper than the polishing redeposition layer, unlike that of Ce and Al, most of which are concentrated in the polishing redeposition layer. The concentration of Fe slightly changes. There is little contamination by the Fe element on the surface after conventional polishing, so the concentration of Fe is almost not decreased after IBE 250 nm. Above all, the TOF-SIMS results reveal that IBE can significantly reduce the surface metal impurity contamination.

### 3.2. Fluorescence Spectra Intensity Evolution

Chemical structural defects, such as an oxygen-deficient center (ODC), are determined to be the main limitations for the improvement in the laser damage resistance of fused silica because of fatigue brittle removal in traditional polishing [22]. Therefore, the fluorescence spectra at different ion beam etched depth surfaces are investigated to characterize the contents of chemical structural defects with the help of typical chemical structural defect peaks. The fluorescence emission spectra of the six samples are excited using a 248 nm laser.

**Figure 3:**
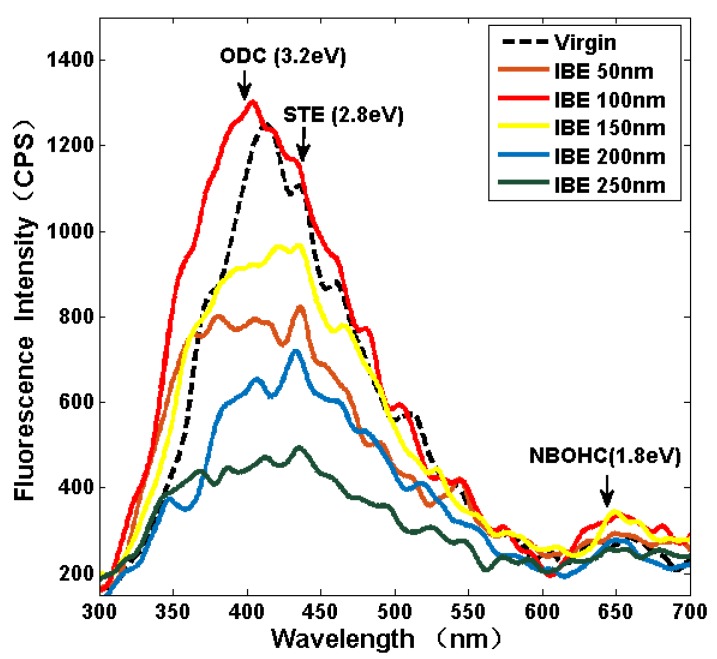
Fluorescence spectra analysis with various IBE depths.

As shown in Figure 3, the arresting peak centered at approximately 400 nm arises from the ODC defects. The nearby peak centered at approximately 443 nm arises from the self-trapped excitation (STE). An unnoticeable peak centered at 650 nm arises from the nonbridging oxygen hole center (NBOHC) [22]. The changes in the ODC and STE intensities are the same. The ODC and STE intensities intensively decrease, whereas the NBOHC intensity rapidly increases when the ion beam etched depth increases to 50 nm. The ODC and STE intensity rapidly increase and present the highest ODC intensity among the others when the IBE depth increases to 100 nm. The NBOHC intensity slightly changes but remains at a high level. The enhancement of characteristic peak intensity may be ascribed to the removal of the polishing redeposition layer. The three types of characteristic peak intensity gradually decrease with the increase in the ion beam etched depth. All the characteristic peak intensities present the lowest level compared with the other etched depth surfaces when the ion etched depth increases to 250 nm. This condition indicates that the shallow IBE with suitable parameters can mitigate chemical structural defects, which is valuable for the improvement in the laser damage resistance of fused silica.

### 3.3. Raman Spectra Intensity Evolution

**Figure 4:**
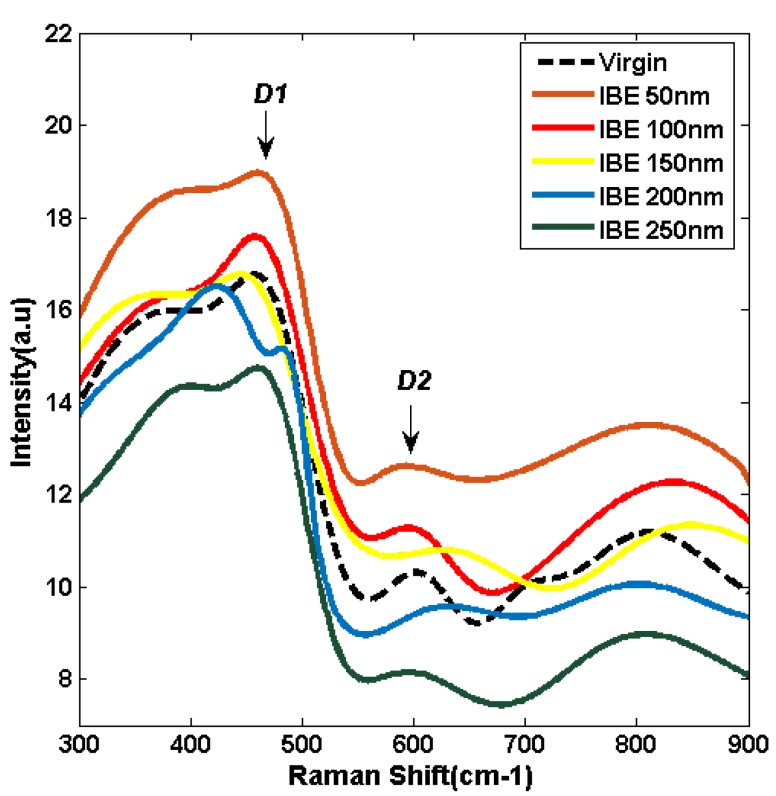
Raman spectra analysis with various IBE depths.

A Bruker Senterra confocal Raman spectrometer is used to characterize the fused silica surface structure changes of six samples during IBE. The Raman spectra of the six samples are excited using a 532 nm laser. The measured Raman spectra of the six samples at different ion beam etched depths are processed through Gauss fitting and are shown in Figure 4. The Raman spectra of fused silica contain a series of broad bands, reflecting the coupled vibrational modes of the silica random network [23].

As illustrated in Figure 4, the arresting peak centered at approximately 490 cm^−1^ (D1) is attributed to the in-phase breathing motions of oxygen atoms in puckered four-membered ring structures, and the peak centered at approximately 605 cm^−1^ (D1) is attributed to the in-phase breathing motions of oxygen atoms in the planar three-membered ring structures [24]. The relative intensities of D1 and D2 Gauss fitting lines initially increase when the ion beam etched depth is 50 nm, indicating that the full nanoscale structural defects in the polishing redeposition layer are removed, and the densification degree increases compared with the virgin surface. The relative intensities of the D1 and D2 Gauss fitting lines gradually decrease with the increase in the IBE depth.

### 3.4. Photothermal Absorption Analysis

Photothermal absorption analysis, which is directly correlated with laser damage precursors on the surface of fused silica optics [25,26], is conducted on the six samples to vividly confirm the laser damage resistance change in different ion beam-etched surfaces. The average photothermal absorption has a high correlation with zero probability LIDT, and there is an exponential attenuation relationship between the surface average photothermal absorption and the zero probability LIDT [27]. The stronger the photothermal absorption signal of the test sample is, the worse the laser damage resistance and LIDT will be. In the testing experiment, the photothermal absorption signal of the six samples is excited using a 355 nm laser. The measurement system is configured to be in reflectance mode. The measurement region is 2.0 mm × 2.0 mm, and the measurement accuracy is 0.1 ppm.

**Figure 5:**
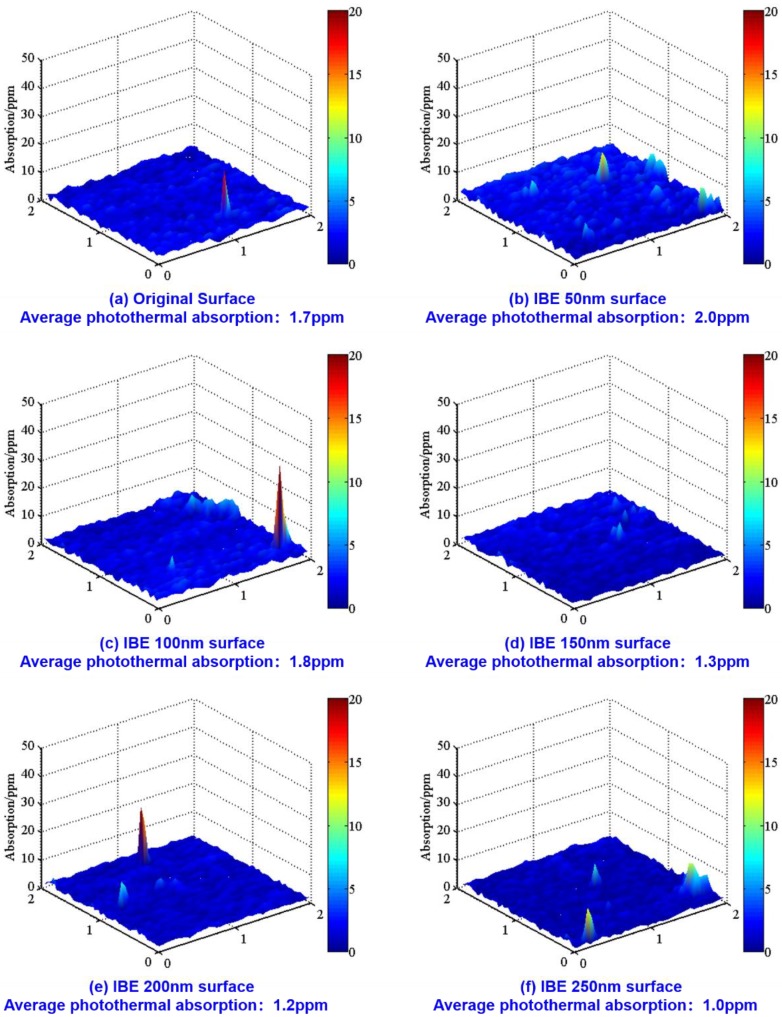
Photothermal absorption distribution in six zones at various IBE depths: (**a**) Original surface: 1.7 ppm; (**b**) IBE 50 nm surface: 2.0 ppm; (**c**) IBE 100 nm surface: 1.8 ppm; (**d**) IBE 150 nm surface: 1.3 ppm; (**e**) IBE 200 nm surface: 1.2 ppm; (**f**) IBE 250 nm surface: 1.0 ppm.

Figure 5 illustrates the photothermal absorption distribution of various IBE depth surfaces in six samples. The overall absorption distribution on the original surface is extremely uniform except for one absorption peak. The initial average photothermal absorption value is 1.7 ppm. At IBE 50 nm, the average absorption value increases to 2.0 ppm, and the number of surface absorption peaks evidently increases, thereby indicating that many damage precursors are exposed and the laser damage resistance decreases. Then, the intensity of the photothermal absorption signal gradually decreases with the increase in the IBE depth. At IBE 250 nm, the photothermal absorption value is 1.0 ppm, which is 41.2% lower than the initial value.

### 3.5. LIDT Test Analysis

LIDT tests are performed on the six samples. A 3 ω Q-switched Nd:YAG laser with 355 nm is used in the LIDT tests. The pulse length τ of the Nd:YAG laser is 7 ns at a repetition rate of 1 Hz. The facula is almost flat Gaussian, and the area of facula with a diameter of 2 mm is about 4.5 mm^2^. R-on-1 testing protocols dictate that a ramping fluence focuses on one region until destruction is evident. Ten test sites are selected randomly to obtain the average R-on-1 threshold for each sample.

**Figure 6:**
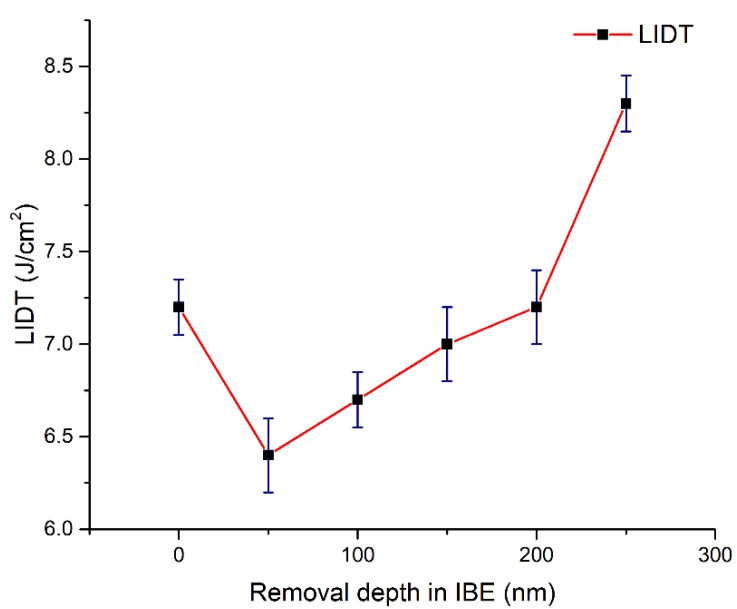
Laser-induced damage threshold (LIDT) evolution at various IBE depths.

The LIDT results at various IBE depths are shown in Figure 6. The LIDT decreases from 7.2 J/cm^2^ of the initial surface to 6.4 J/cm^2^ of the 50 nm IBE depth. Then, the LIDT gradually goes up with the increase in the IBE depth. At IBE 250 nm, the LIDT is 8.3 J/cm^2^, which is 15.2% higher than the initial value. The LIDT test results reveal that IBE can significantly improve the LIDT of fused silica in proper etched depth.

## 4. Discussion

IBE can effectively remove or mitigate surface fracture defects, such as brittle scratches, to improve the laser damage resistance of fused silica. In the previous research, this passivation phenomenon on damage precursors during IBE has been investigated [16,18]. IBE removes the fused silica material and maintains the surface roughness with the help of the ion sputtering effect. Poor surface roughness enhances the small-scale self-focusing effect, resulting in the concentration of local light intensity on the sample surface. Local light intensity is correlated to laser damage resistance, and the higher the light intensity, the better the laser damage resistance will be. Therefore, the fused silica optics in high-power laser systems must have supersmooth surfaces to completely reduce the small-scale self-focusing effect [16]. On the basis of the results in Section 3, the impurity contamination introduced by traditional mechanical manufacturing (such as grinding, lapping, and polishing) is reduced when the material is gradually removed during IBE. Compared with the original surface, the characteristic peak intensity of the IBE surface chemical structure is significantly reduced. The influences of the nanoscale intrinsic surface characteristics on laser damage resistance are analyzed and investigated.

After traditional polishing, many impurity contaminations are concentrated on the polishing redepositing layer, which is an important factor limiting the increase in LIDT. In the previous research, these nanoscale impurity contaminations that originated from polishing absorb sub-band gap light and cause a significant reduction in LIDT [21,28]. On the basis of the TOF-SIMS results in Section 3.1, the reduction in impurity contamination concentration indicates that IBE has a cleaning effect on metal impurity contaminations. For IBE 250 nm, the concentrations of metal impurities, which may cause strong absorption on the UV and low laser damage performance, significantly decrease. Thus, laser damage resistance is immensely improved through IBE removal at 250 nm.

A previous study showed that some chemical structural defects evolve into high threshold damage precursors and induce laser damage initiation at fluences and intensities at the damage threshold of 10 J/cm^2^ [5]. The fluorescence spectra analysis results in Section 3.2 indicate that the original surface contains a large number of defects, such as ODC, STE, and NBOHC. These defects are widely distributed on the sample surface at the atomic scale, thereby weakening the fused silica network structure and reducing the damage resistance of the material. Chemical structural defects easily induce multiphoton absorption, and initial free electron is generated through ionization. The free electron density increases until a breakdown occurs in the material when the defects continue to absorb laser energy. All the characteristic peak intensities of chemical structural defects present the lowest level among other etched depth surfaces when the ion etched depth increases to 250 nm. The passivation phenomenon during IBE is beneficial to reduce the atomic-scale defects of fused silica. Therefore, we assume that the laser damage resistance is improved by IBE. The Raman spectra analysis results in Section 3.3 suggest that the ring structures on the fused silica surface change during IBE. The relative intensities of D1 and D2 can reflect the density of the fused silica surface. The surface densification degree and the relative intensities of D1 and D2 increase when the polishing redeposition layer is removed. However, a subsurface densification layer remains, where its density is larger than the intrinsic fused silica surface because of the positive pressure of traditional polishing. The surface densification is alleviated, and the relative intensities of D1 and D2 decrease when IBE removes the subsurface deformation layer. The properties of the new surface approach the inherent nature of fused silica when the IBE removes the subsurface deformation layer.

**Figure 7:**
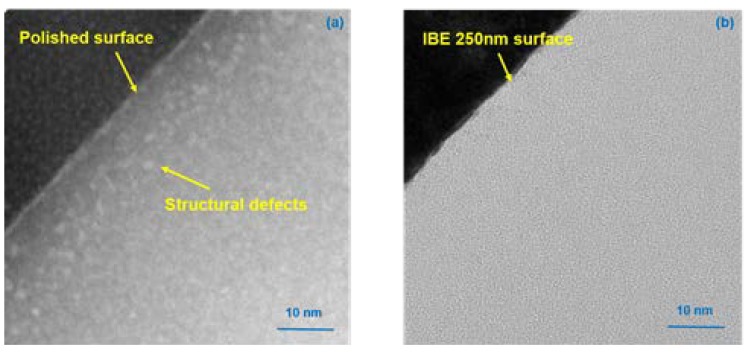
Subsurface layer in HRTEM: (**a**) Traditional polished surface (sample 0#): the structural defect layer formed during traditional polishing; (**b**) IBE 250 nm surface (sample 5#): a near-perfect subsurface is obtained with IBE.

To provide additional details on the evolution of chemical structural defects during IBE, HRTEM experiments are conducted to observe the subsurface morphology evolution before and after IBE (samples #0 and #5). Detailed material quality beneath the surface in IBE can be obtained. The subsurface morphologies in HRTEM of different ion beam etched depths are compared, as shown in Figure 7. Interference occurs between the beam, diffraction beam, and center transmitted electron when they pass through the sample to be tested, thereby forming the contrast of HRTEM figures. The imaging contrast is insufficient and cannot clearly describe each atomic structure because fused silica is an amorphous phase material. However, the characteristics of nanoscale structural defects can be ascribed to the different material properties in the fused silica subsurface. For the traditional polished sample surface (sample #0), Figure 7a illustrates that the characteristic of the subsurface (0–20 nm depth), where visible structural defects (1–2 nm in size) are uniformly distributed, is clearly distinguished from the fused silica substrate. During traditional mechanical manufacturing (such as grinding, lapping, and polishing), considerable structural vacancies are introduced into the subsurface of the optics. Compared with the inherent nature of fused silica, the density of the polishing redeposition layer is lower, which is consistent with the relative intensity change of D1 and D2 in the Raman spectra analysis. The interface between the subsurface layer full of nanoscale structural defects and the matrix is insufficiently distinct. Considerable structural defects are embedded in the subsurface layer of traditional polished fused silica. Under sufficient fluence ultraviolet lasers, structural defects evolve into damage precursors and induce laser damage. After 250 nm IBE treatment (sample #5), the distribution of material properties is consistent, and no structural defects are found, as shown in Figure 7b, thereby indicating that the subsurface layer filled with structural defects is completely removed by IBE. The properties of the new surface approach the intrinsic surface of fused silica.

On the basis of the results in Figure 5 and Figure 6, the influences of various nanoscale damage precursors on the laser damage resistance of fused silica optics should be comprehensively considered. The LIDT of the initial surface is 7.2 J/cm^2^ and the average photothermal absorption value is 1.7 ppm. At IBE 50 nm, the LIDT decreases and the number of surface absorption peaks evidently increases, thereby indicating that many damage precursors are exposed and that the laser damage resistance decreases. This result corresponds to the test results in HRTEM. The arrangement image of the subsurface material particles in Figure 7a in HRTEM proves that huge amounts of structural defects are embedded into the original subsurface. Structural defects are mainly distributed in the area with 20 nm depth. Thus, a large number of structural defects are exposed after the removal of the polishing redeposition layer at IBE 50 nm, thereby promoting the surface average absorption and worsening the surface laser damage resistance.

When the IBE depth increases from 50 to 250 nm, the intensity of photothermal absorption gradually decreases. The LIDT test results proves that the surface laser damage resistance is effectively enhanced during IBE, which is consistent with the evolution results of impurity contamination, fused silica surface structural defects, and surface photothermal absorption. The surface metal impurity concentration evidently decreases with the increase in the IBE depth. For the chemical structural defects, the concentrations of ODC, STE, and NBOHC decrease. IBE reduces the relative intensities of D1 and D2 lines and alleviates the surface densification caused by traditional polishing. The decrease in photothermal absorption results and the improvement in the LIDT test results both indicate that the nanoscale subsurface defects containing impurity contamination and structural defects in the subsurface play a crucial role in laser damage resistance. IBE technology is an effective method of removing these nanoscale defects and enhancing the laser damage resistance of fused silica. It should be noted that the advantage of the IBE over HF etching is not obvious. The LIDT increasing in [7] through HF acid chemical etching occurs from an initial value of about 16 J/cm^2^ to about 30 J/cm^2^. Alternative effects lead to the insufficient enhancement in LIDT. Most likely, residual impurity contamination and the ion bombardment from IBE should be responsible. For IBE 250 nm, there is some impurity contamination by the Fe, Al, and Ca elements on the surface, thereby restricting the enhancement of LIDT. Increasing the removal depth of IBE gradually is helpful to mitigate the impurity contamination and raise the LIDT. In addition, a disturbed layer containing lattice dislocation, vacancy, and displacement is formed as a result of Ar^+^ ion bombardment, which is the subsurface damage that is different from the main material on the dielectric constant [29,30]. The volume inhomogeneities of the disturbed layer induced by IBE cannot be neglected. As a result of it, radiation scattering and phonon generation can occur; in addition, laser radiation can be reflected and focused or interfere with the incident wave, which causes laser damage and worsens the LIDT. IBE provides a new way to mitigate the nanoscale damage precursors of fused silica, but the parameters (such as beam energy, beam current, and incidence angle) in this postprocessing need to be improved to decrease the disturbed layer depth and realize the full potential of raising the LIDT. Finally, the removal rate of fused silica in IBE is relatively low compared with MRF and HF acid etching. In MRF, the removal rate of fused silica is about 0.1 mm^3^/min, and HF acid chemical etching is a type of surface global postprocessing, whose removal rate on fused silica is about 20 nm/min. IBE will often be combined with other postprocessing to remove nanoscale damage precursors quickly and efficiently in future applications.

## 5. Conclusions

In this paper, the experiments are focused on the evolution of several types of nanoscale damage precursors in shallow IBE, which is significant for determining the inherent characteristics of nanoscale damage precursors on the surface laser damage resistance.

IBE can reduce the impurity contamination and chemical structural defects by increasing the etched depth. IBE can eliminate the polishing redeposition layer and weaken the surface densification. The HRTEM experiments characterize the subsurface structural defects before and after IBE in detail. After IBE at 250 nm, a nonfracture subsurface quality can be obtained with IBE. The laser damage performance in IBE is significantly improved on the basis of these favorable factors. This postprocessing technique needs to be improved and could be used in conjunction with HF acid chemical etching to realize the full potential of raising the LIDT. This work can serve as a reference in understanding the effects on nanoscale damage precursor evolution in conducting IBE technologies on fused silica.
